# Detection of Venom after Antivenom Is Not Associated with Persistent Coagulopathy in a Prospective Cohort of Russell's Viper (*Daboia russelii*) Envenomings

**DOI:** 10.1371/journal.pntd.0003304

**Published:** 2014-12-18

**Authors:** Kalana Maduwage, Margaret A. O'Leary, Fiona E. Scorgie, Seyed Shahmy, Fahim Mohamed, Chandana Abeysinghe, Harindra Karunathilake, Lisa F. Lincz, Christeine A. Gnanathasan, Geoffrey K. Isbister

**Affiliations:** 1 School of Medicine and Public Health, University of Newcastle, Callaghan, New South Wales, Australia; 2 Faculty of Medicine, University of Peradeniya, Peradeniya, Sri Lanka; 3 Department of Clinical Toxicology and Pharmacology, Calvary Mater Newcastle, Newcastle, New South Wales, Australia; 4 Hunter Haematology Research Group, Calvary Mater Newcastle, Newcastle, New South Wales, Australia; 5 South Asian Clinical Toxicology Research Collaboration (SACTRC), Faculty of Medicine, University of Peradeniya, Peradeniya, Sri Lanka; 6 Clinical Pharmacology and Toxicology Group, Professorial Medicine Unit, The Prince of Wales Clinical School, University of New South Wales, Sydney, New South Wales, Australia; 7 District Hospital Hingurakgoda, Hingurakgoda, Sri Lanka; 8 General Hospital Matara, Matar, Sri Lanka; 9 Department of Clinical Medicine, Faculty of Medicine, University of Colombo, Colombo, Sri Lanka; Liverpool School of Tropical Medicine, United Kingdom

## Abstract

**Background:**

Venom recurrence or persistence in the circulation after antivenom treatment has been documented many times in viper envenoming. However, it has not been associated with clinical recurrence for many snakes, including Russell's viper (*Daboia* spp.). We compare the recovery of coagulopathy to the recurrence or persistence of venom in patients with Russell's viper envenoming.

**Methodology/Principal Findings:**

The study included patients with Russell's viper (*D. russelii*) envenoming presenting over a 30 month period who had Russell's viper venom detected by enzyme immunoassay. Demographics, information on the snake bite, and clinical effects were collected for all patients. All patients had serum collected for venom specific enzyme immunoassay and citrate plasma to measure fibrinogen levels and prothrombin time (international normalised ratio; INR). Patients with venom recurrence/persistence were compared to those with no detectable recurrence of venom. There were 55 patients with confirmed Russell's viper envenoming and coagulopathy with low fibrinogen concentrations: 31 with venom recurrence/persistence, and 24 with no venom detected post-antivenom. Fibrinogen concentrations increased and INR decreased after antivenom in both the recurrence and non-recurrence patients. Clinical features, laboratory parameters, antivenom dose and length of hospital were similar for both groups. Pre-antivenom venom concentrations were higher in patients with venom recurrence/persistence with a median venom concentration of 385 ng/mL (16–1521 ng/mL) compared to 128 ng/mL (14–1492 ng/mL; p = 0.008).

**Conclusion:**

Recurrence of Russell's viper venom was not associated with a recurrence of coagulopathy and length of hospital stay. Further work is required to determine if the detection of venom recurrence is due to the venom specific enzyme immunoassay detecting both venom-antivenom complexes as well as free venom.

## Author Summary

Snakebite is a major public health problem and understanding the effectiveness of antivenom is essential to improving health outcomes. The measurement of venom in blood has been used to assess the effectiveness of antivenom. The absence of venom post-antivenom indicating that sufficient antivenom has been given, and the persistence or recurrence of venom indicating that insufficient antivenom has been given. There are numerous reports of venom recurrence with viper bites, including Russell's viper bites. However, it remains unclear if venom recurrence is always an indicator of inadequate antivenom and recurrence of clinical envenoming. In this study, we compare patients with and without the persistence or recurrence of venom who develop coagulopathy after Russell's viper bites. There was no difference in the recovery of the coagulopathy between the two groups of patients demonstrating that for Russell's viper envenoming, venom recurrence or persistence was not associated with the recurrence or persistence of clinical effects such as coagulopathy. Patients with detectable venom after antivenom did have higher pre-antivenom venom concentrations. Further investigation is required to interpret venom concentrations post-antivenom.

## Introduction

Snake envenoming is a major public health issue in many resource poor countries in the rural tropics [1]. Understanding the underlying pathophysiology of snake envenoming and the effect of antivenom is essential to improving health outcomes. An important part of investigating snake envenoming is the detection and measurement of venom in human sera, which confirms the type of snake (diagnosis) as well as assessing the efficacy of antivenom. As such, venom concentrations are measured before and after antivenom and the absence of venom in blood post-antivenom has been interpreted to mean that sufficient antivenom has been administered and envenoming will resolve. This has been used as an important end-point in recent studies in Australia showing that one vial of antivenom is sufficient for the treatment of all Australian elapids [2]. The persistence or recurrence of venom after antivenom administration has been interpreted as insufficient antivenom being administered and there being ongoing envenoming.

The phenomenon of persistent or recurrent venom in patients following antivenom administration has been reported many times for a number of snakes, including Russell's viper (*Daboia russelii* and *D. siamensis*) [3]–[8], Malayan Pit viper (*Calloselasma rhodostoma*) [9], [10], Carpet viper (*Echis ocellatus*) [11], (*Echis pyramidum*) [12], Western Diamond back rattle snake (*Crotalus atrox*) [13]–[15], and Lancehead vipers (*Bothrops* species) [16]. Most studies have been conducted in Russell's viper because of the importance of this snake in South Asia, and have reported recurrence rates ranging from 7 to 95% [3]–[8], [17]. To date, the recurrence of venom detected by EIA in sera post-antivenom has been interpreted as a failure of the initial antivenom dose to be effective or sufficient. Most experts usually suggest that there is ongoing absorption of venom from the site of the bite to the systemic circulation due to the large dose of venom injected by vipers [4]–[6]. Although it is often assumed and stated that there is ongoing clinical envenoming or recurrent clinical envenoming associated with the detection of venom post-antivenom, this has never been conclusively proven. In the case of Russell's viper envenoming it is not clear whether there is a recurrence of coagulopathy with the recurrence of venom in the circulation.

Russell's viper venom contains factor X and factor V activators which result in the activation of the clotting pathway manifesting as venom induced consumption coagulopathy (VICC) [18], [19]. VICC in Russell's viper envenoming is characterised by a prolonged prothrombin time (PT) or international normalised ratio (INR), decreased levels of fibrinogen, decreased levels of factor V, decreased levels of factor X, and elevated d-Dimer concentrations [6], [20]–[22]. Once antivenom is administered there is a resolution of VICC with normalising of the clotting function times and replenishing of the clotting factors including a gradual increase in fibrinogen levels.

We hypothesized that if sufficient antivenom had been administered, then there would be an improvement in clotting function despite persistent or recurrent venom being detected using EIA. The aim of this study was to compare the recovery of VICC in patients with and without venom recurrence/persistence. The recovery of VICC was assessed primarily by the recovery of fibrinogen levels over time.

## Methods

This was a prospective observational cohort study of definite Russell's viper (*Daboia russelii*) bites that compared patients with and without recurrence or persistence of venom post-antivenom. It was conducted as part of a large cohort study of patients with snakebites presenting to the Base Hospital Chilaw in Central West Sri Lanka [23]. The study was approved by the Ethical Review Committee, Faculty of Medicine, University of Colombo and Faculty of Medicine, University of Peradeniya, Sri Lanka. All patients gave written and informed consent for the collection of clinical data and blood samples.

### Patients

Patients over 13 years of age who presented with Russell's viper envenoming and coagulopathy from January 2007 to July 2009 were included in the study. Cases were only included if Russell's viper venom was detected in the patients' serum with the Russell's viper venom-specific EIA. We included only patients who had citrate and serum samples collected before antivenom administration, and who had at least three samples collected up to 24 hours after antivenom. The median number of samples collected from the patients was 5 (Range: 3 to 10).

### Data collection

The following data were collected from patients prospectively: age and sex, time of the snakebite, clinical effects (local effects, coagulopathy, systemic bleeding [haematemesis, bleeding gums or haematuria], neurotoxicity [ptosis, ophthalmoplegia] and non-specific systemic symptoms), antivenom treatment (timing and dose) and hospital length of stay. Additional blood samples were collected from all patients on admission and then for at least 24 hours after antivenom treatment. Blood was collected in citrated tubes for coagulation studies and in serum tubes for venom-specific EIA. All samples were immediately centrifuged, aliquotted and frozen at −20°C and then transferred to a −80°C freezer within 2 weeks of collection until the completion of the study. All patients received Indian polyvalent snake antivenom manufactured by VINS Bioproducts Limited (batch number: ASV 42C/06, 1030) or BHARAT (batch number: 5346KD4, LY 55/05, LY 32/04, A5307035) Serum and Vaccines Limited, India. Both are equine F(ab′)_2_ antivenoms.

### Venom specific enzyme immunoassays (EIA)

Russell's viper venom concentrations were measured in serum samples by sandwich EIA which has previously been described [23]–[26]. In brief, polyclonal IgG antibodies were raised against Russell's viper (*D. russelii*) venom in rabbits as previously described [27]. These were bound to the microplates as well as being conjugated to biotin for a sandwich EIA with the detecting agent streptavidin-horseradish peroxidase. All samples were measured in triplicate, and the averaged absorbance converted to a concentration by comparison with a standard curve based on serial dilutions of venom using a sigmoidal curve.

### Fibrinogen and prothrombin time (PT) assays

Prothrombin times (PT), international normalised ratio (INR) and fibrinogen concentrations were measured in platelet free citrated plasma samples. All assays were performed using standard coagulometric methods on the Behring Coagulation System (Siemens, Marburg, Germany) or Sysmex CA-1500 coagulation analyser (Siemens, Marburg, Germany), respectively. Briefly, the PT was determined by mixing patient plasma and Innovin reagent (Dade Behring Inc, USA) and the time taken for fibrin clot formation was measured. The INR was calculated from the PT according to the recommended formula. The fibrinogen concentration was determined by mixing a 1∶10 dilution of patient plasma in Owrens Veronal Buffer, in a 2∶1 ratio with Dade Thrombin Reagent (Siemens Healthcare Diagnostic Inc, USA) and measuring the time to fibrin clot formation. The fibrinogen concentration was then determined from the time to clot formation according to a standard curve of serially diluted standard human plasma in g/L.

### Data analysis

Patients with venom detected after the administration of antivenom, whether after an initial decrease in venom concentrations (recurrence) or no initial decrease in venom concentration (persistence), were defined as patients with venom recurrence/persistence. These patients were then compared to patients where venom was never detected after the administration of antivenom. Time to the recovery of fibrinogen concentration and INR, pre-antivenom venom concentrations, clinical effects (coagulopathy [INR>1.5], neurotoxicity [ptosis], systemic bleeding and local envenoming), number of antivenom doses administered and length of hospital stay, were compared between the venom recurrence or persistent group and the group of non-recurrence patients.

Continuous variables (venom concentrations, time to fibrinogen and INR recovery, and length of hospital stay) are reported as median values with interquartile ranges (IQR) and ranges. Continuous variables were compared with the Mann-Whitney test (non-parametric). All analyses and graphics were done in GraphPad Prism version 6 for Windows, GraphPad Software, San Diego California USA, www.graphpad.com.

## Results

**Figure 1:**
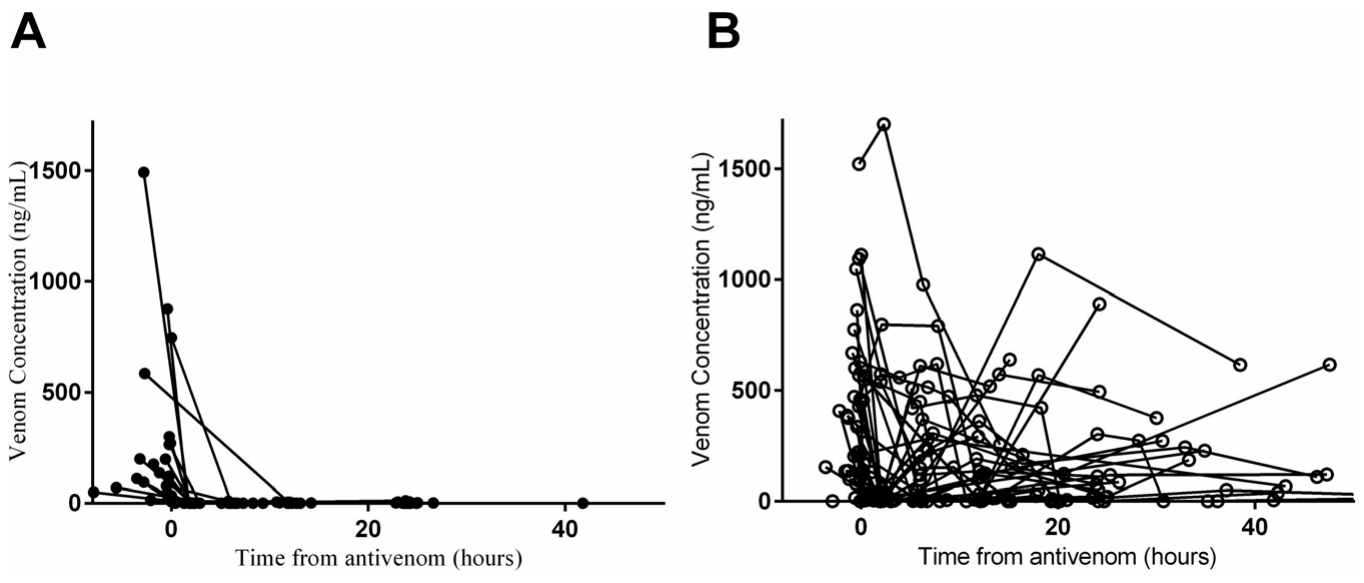
Concentrations of Russell's viper venom (ng/ml) versus time in the patients without recurrence (Panel A) compared to patients with persistence/recurrence (Panel B).

Russell's viper envenoming was confirmed in 173 patients by EIA, but only 55 patients were included in the analysis due to adequate numbers of blood samples. In 24 patients, venom was not detected in serum after the administration of antivenom (Table 1; Fig. 1A). In 31 patients there was recurrence or persistence of Russell's viper venom after antivenom administration (Table 1; Fig. 1B).

**Table 1:** Comparison of patients with Russell's viper envenoming and venom recurrence/persistence and non-recurrence of venom, including clinical features of envenoming, coagulopathic parameters, treatment and outcomes.

	Non-recurrence	Venom recurrence
Number of patients	24	31
Male∶female	17∶7	26∶5
Median age (years) (range)	38 (19 to 70)	35 (16 to 82)
Local envenoming	16	20
Systemic bleeding	1	5
Neurotoxicity (ptosis)	9	25
Type of antivenom, VINS: BHARAT	5: 17	11: 18
Median lowest fibrinogen g/L	0.8 (0.01–1.3)	0.5 (0.01–1.5)
Median highest INR	4.8 (2.6–13)	6.4 (2–13)
Median pre antivenom RV venom concentration ng/ml (range)	128 (14–1492)	385 (16–1521)
Median dose of antivenom (vials)	10	10
Number of patients received repeat antivenom doses	2	14
Median length of hospital stay (days)	2 (1 to 5)	3 (1 to 9)

INR, International normalised ratio.

**Figure 2:**
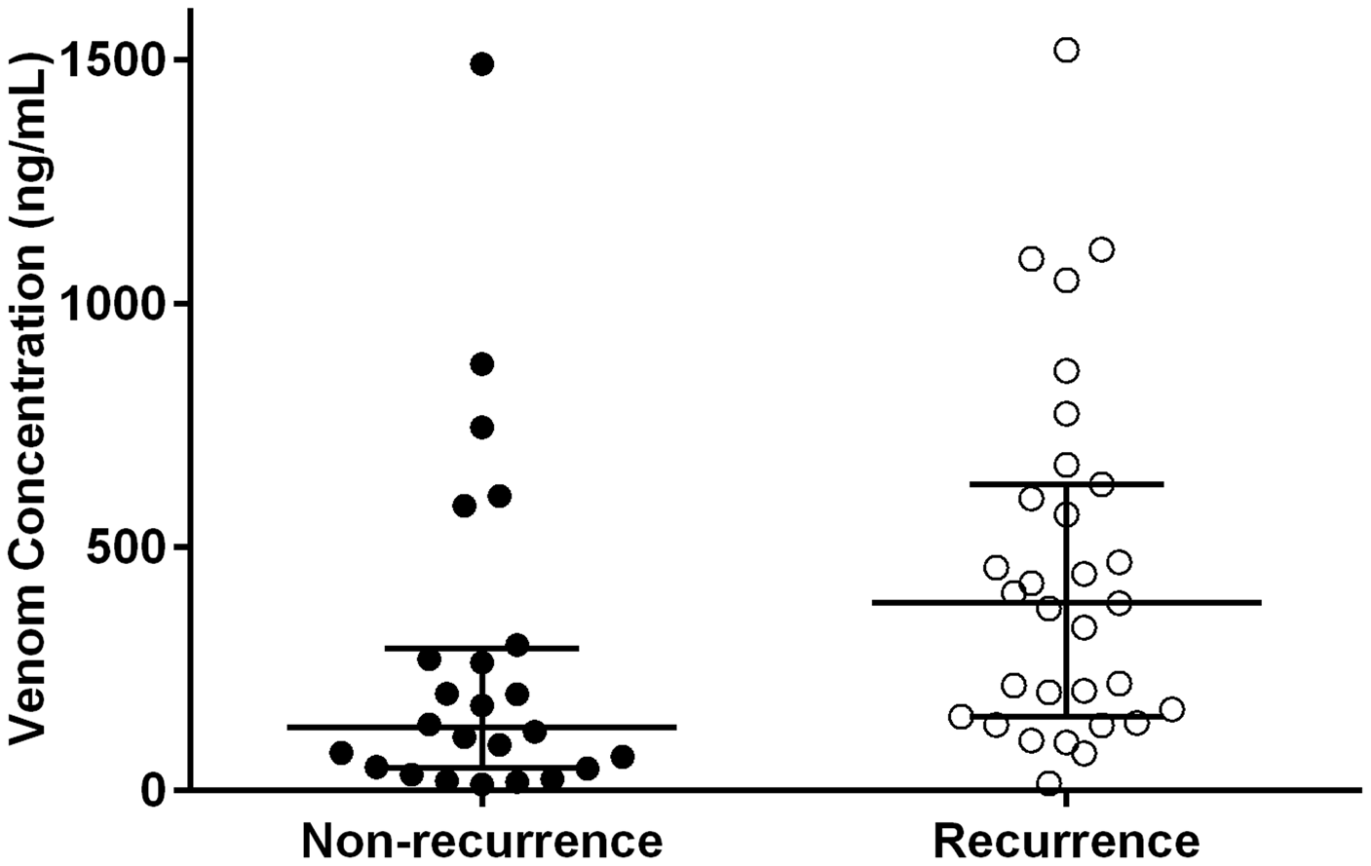
Scatterplot of the pre-antivenom Russell's viper venom concentrations (ng/ml) in the patients without recurrence (filled circles) compared to patients with persistence/recurrence (open circles).

All 55 patients developed VICC with an abnormal INR and low or undetectable fibrinogen in the pre-antivenom or admission blood sample. Clinical features, coagulopathic parameters, pre-antivenom venom concentrations, antivenom dose and length of hospital stay are shown in Table 1. The severity of the coagulopathy as determined by the median highest INR and median lowest fibrinogen, was similar for patients with recurrence or persistence of venom versus patients without recurrence (Table 1). Pre-antivenom venom concentrations were significantly higher in patients with venom recurrence or persistence with a median venom concentration of 385 ng/mL (16 to 1521 ng/mL) compared to patients without recurrence/persistence with a median venom concentration of 128 ng/mL (14 to 1492 ng/mL; p = 0.008; Fig. 2).

**Figure 3:**
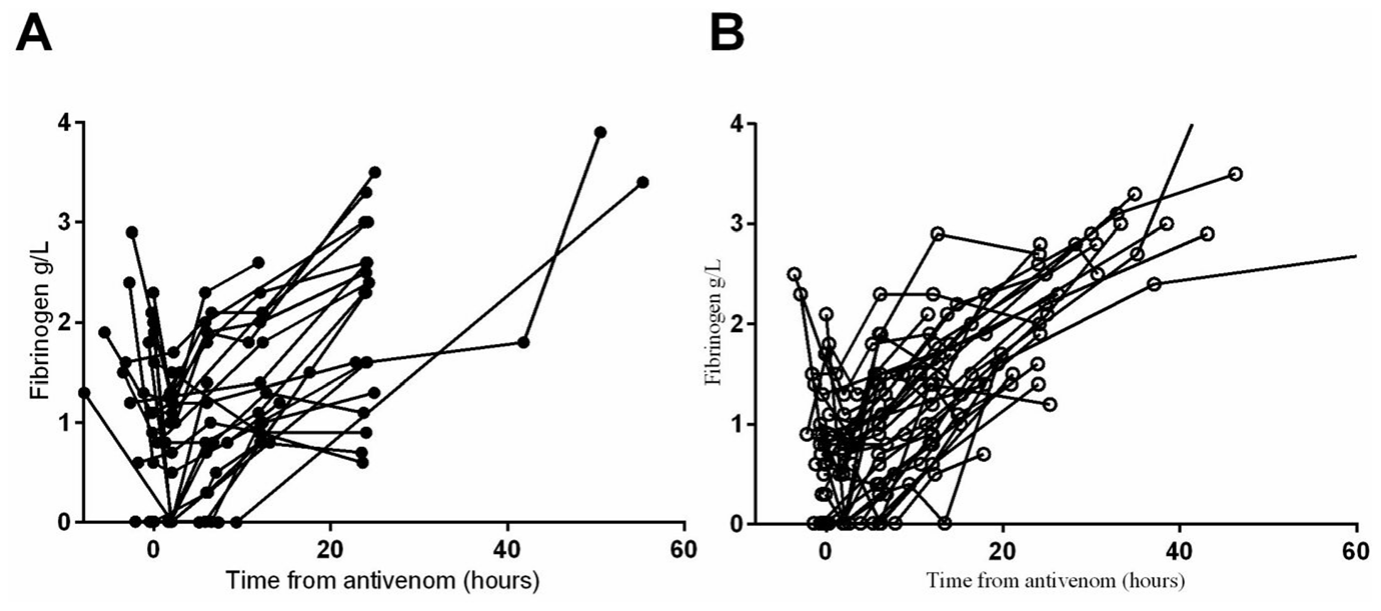
Fibrinogen levels (g/L) versus time in Russell's viper envenoming comparing patients without recurrence (Panel A) to patients with persistence/recurrence (Panel B).

**Figure 4:**
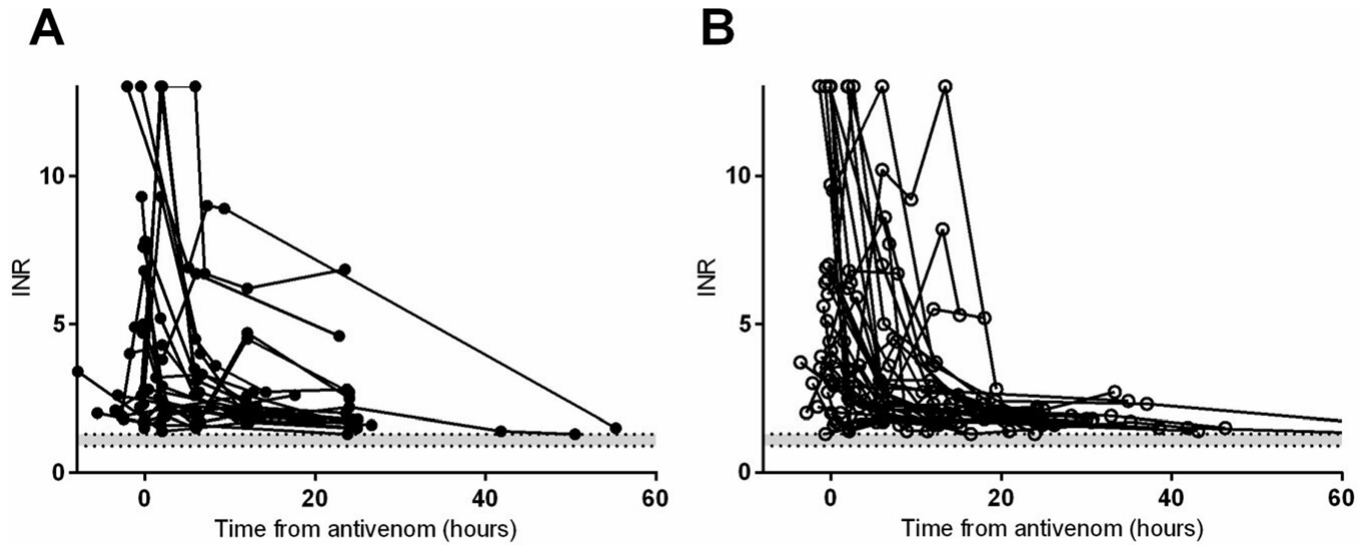
International normalised ratio (INR) versus time in Russell's viper envenoming comparing patients without recurrence (Panel A) to patients with persistence/recurrence (Panel B).

The median time to recovery of the fibrinogen concentration to 1 g/L was 11.5 hours (0.3 to 34.9 h) in patients with venom recurrence/persistence compared to 12.3 hours (1.8 to 55.3 h) in patients without venom recurrence which was not significantly different (p = 0.75; Figs. 3A and 3B). Similarly, the median time to correct the INR to less than 2 was 11.8 hours (0.3 to 32.9) for patients with venom recurrence or persistence compared to 12.3 hours (5.8 to 55.3 h) in patients without recurrence which was not statistically significant (p = 0.21;Figs. 4A and 4B).

Patients in both groups were treated with either VINS or BHARAT Indian polyvalent antivenoms and a median of 10 vials of antivenom was given in both groups. However, 14 patients in the recurrence group had multiple doses of antivenom and only two patients in the non-recurrence group had repeated doses of antivenom. Administration of the second dose of antivenom in these patients was based on a positive whole blood clotting test 20 minutes (WBCT 20). However, in ten out of 14 patients in the recurrence group who had a second dose of antivenom, the coagulopathy had already resolved with a normal INR and fibrinogen, not consistent with the positive WBCT 20 done at the time. The remaining two patients in the recurrence group, and two patients in the non-recurrence group who had additional antivenom, were in the recovery phase of the coagulopathy (S1 Figure). The median length of hospital stay was 3 and 2 days in recurrence and non-recurrence groups, respectively, which was not statistically significant (Table 1).

## Discussion

This study shows that the measurement of venom post-antivenom, either seen as the persistence of venom or the recurrence of venom, was not associated with ongoing coagulopathy in Russell's viper bites. The time to recovery of fibrinogen, the time to recovery of INR, and length of hospital stay were similar in both the venom recurrence/persistence group and the non-recurrence group. However, pre-antivenom venom concentrations were higher in recurrence/persistence group compared to the non-recurrence group.

Recurrence of coagulopathy associated with the Russell's viper venom recurrence has been described previously in single case reports [4], [5], [10]. All of these studies have used the WBCT 20 or methods measuring clot quality to provide evidence for recurrence of coagulopathy. In addition, the so-called venom recurrence was recognised many months after the patient was treated and discharged, when the venom assays were done. Ariaratnam et al. selectively describe a single case from a study of 35 patients where the WBCT 20 became abnormal after antivenom administration, thus prompting a second dose of antivenom. This was later found to coincide with the measurement of venom recurrence. However, venom recurrence occurred in the majority of the patients in their study and in 11 patients given only a single dose of 2 g antivenom (PolongaTab), they state that all patients had recurrence and persistence of venom for a mean time of 72 hours, despite all having immediate reversal of systemic envenoming including coagulopathy [4]. A different interpretation of the data is that Ariaratnam et al show that recurrent venom antigenaemia is not associated with coagulopathy in the majority of their cases, similar to our study, and in one case only there was an abnormal WBCT 20 at the time of the recurrence. This single abnormal WBCT 20 was potentially an error and the investigators did not confirm coagulopathy with other clotting studies. Lack of sensitivity of WBCT 20 for the detection of Russell's viper coagulopathy has been recently demonstrated [28], and is the probable explanation for the recurrence of coagulopathy in previous reports [4], [10] as well as the 10 abnormal WBCT 20 that occurred in our study.

The case reported by Theakston is a single case in which the reported venom concentrations were very low (<15 ng/mL) compared to ours and other studies measuring Russell's viper venom [3], [4], [6]–[8], [29]. Ho et al report a number of cases but there is no correlation between the recurrence of venom and the recurrence of an abnormal WBCT 20 (Table 3; Ho et al.), except in one patient. This again suggests that recurrent coagulopathy did not occur with the measurement of venom recurrence [10]. Recurrence of coagulopathy in Russell's viper envenoming has not been described in any of the studies that used formal laboratory coagulation studies to assess the coagulopathy including fibrinogen concentration or clotting factor assays [6], [21], [30], [31].

Venom recurrence has been documented with other vipers [10]–[13], [15], [16]. In a number of cases this is similar to previous studies of Russell's viper where there is little evidence to support recurrence of clinical envenoming. However, for some snakes there appears to be recurrence of clinical and/or laboratory envenoming at the same time as there is venom recurrence, but in these cases the recurrence appears to occur days after the bite, unlike the 12 to 24 hours in our study [15]. This is demonstrated clearly in a phase II clinical trial by Boyer et al of American vipers where the venom recurrence occurs approximately one week after the administration of antivenom in patients given Fab antibodies compared to no recurrence in those given F(ab′)_2_ antibodies [15].

The recurrence reported in American viper envenoming appears to be different to that seen with reports from Asian and African vipers. It occurs much later and has only been recognised with the change from an F(ab′)_2_ antivenom to an Fab antivenom. The recurrence is thought to be due to the rapid clearance of Fab and ongoing persistence of venom. Persistent coagulopathy has been reported in North American Crotalid envenoming for many years [32]–[37] and is thought to be due to slow absorption of venom. Therefore, the mismatch of Fab antivenom pharmacokinetics with rapid elimination and the slow venom absorption are the likely reason for the recurrence of coagulopathy. The recent study by Boyer et al confirms this [15].

It has always been assumed that venom specific EIA only detects free venom. It reasonably follows that the presence of free venom detected after antivenom administration means that insufficient antivenom has been given. A recent study has shown that in fact the traditional venom specific EIA (as originally developed by Theakston [38]) can detect bound venom or venom-antivenom (VAV) complexes under certain conditions [39]. If there are high concentrations of antivenom such that venom molecules are surrounded by antivenom and there is excess free antivenom, no venom is detected by the traditional venom EIA. However, at lower concentrations of antivenom (lower ratio of antivenom to venom), where every venom component is not completely surrounded by antivenom but attached to at least one antivenom molecule or antibody on average, the traditional venom EIA will detect bound venom or VAV complexes (see figure 1 O'Leary and Isbister 2014 [39]). It was therefore proposed that the detection of venom in sera post-antivenom may not mean that insufficient antivenom has been administered, because the assay is detecting bound venom. This is not surprising since it is well known that the assay for digoxin will detect digoxin bound to digoxin antibodies after the administration of digoxin antibodies [40], [41]. This VAV assay was developed with F(ab′)_2_ antivenom [39].

We found that patients with persistence or recurrence of venom had significantly higher pre-antivenom venom concentrations (Table 1). This is consistent with the hypothesis that the venom assay detects VAV complexes, because patients with a high venom load will have a lower ratio of antivenom to venom molecules and therefore, VAV complexes that can be detected (Fig. 1B). In contrast, patients with lower concentrations of Russell's viper venom will have the venom molecules completely surrounded by the same dose of antivenom, so VAV complexes cannot be detected and there is no apparent venom recurrence with EIA (Fig. 1A). Clearly, if there is insufficient antivenom there will be persistence of free venom in addition to VAV complexes. Further assays need to be developed to help distinguish free venom from bound venom to confirm that there is no free venom present in samples collected post-antivenom in our study.

An unusual finding in the study was that repeat dosing of antivenom was more common in the group with venom recurrence or persistence. This could be interpreted to mean that patients with recurrence were given further antivenom because of suspected recurrent or ongoing envenoming or coagulopathy. This is partly correct because these patients still had an abnormal WBCT 20 which was the likely reason for a second dose of antivenom being given. However, all of these patients had a normal INR or an improving INR showing that the coagulopathy was in fact resolving, and that the WBCT 20 was incorrect.

Another difference between to two groups was that patients with persistence or recurrence were more likely to have neurotoxicity and systemic bleeding. This is again consistent with this group having higher venom concentrations and it therefore making sense that they had more severe envenoming. Both systemic bleeding and neurotoxicity (mostly ptosis) do not rapidly reverse with antivenom, so their persistence after antivenom does not indicate recurrent envenoming. In fact, this also further explains why repeat doses of antivenom were given in this group because of the common misconception that neurotoxicity will immediately resolve with antivenom, and if it hasn't, the patient needs more antivenom.

Our study shows that the recurrence or persistent of venom post-antivenom does not necessarily mean that insufficient or inefficacious antivenom has been given. For the doses of antivenom given in these patients with Russell's viper envenoming, sufficient antivenom had been given and the coagulopathy was recovering. Further work is required to clarify the best measures of antivenom efficacy in vivo.

## Supporting Information

S1 Figure Fibrinogen levels (g/L) versus time in the two patients in the recurrence group and two patients in the non-recurrence group who had additional antivenom and were in the recovery phase of the coagulopathy.
(JPG)

S1 Checklist STROBE checklist.
(DOCX)
