# Supplementary figures and images for: Detection of Venom after Antivenom Is Not Associated with Persistent Coagulopathy in a Prospective Cohort of Russell's Viper (Daboia russelii) Envenomings
Source: PLoS Negl Trop Dis. 2014 Dec 18;8(12):e3304. doi: 10.1371/journal.pntd.0003304 (PMC4270487; doi:10.1371/journal.pntd.0003304)

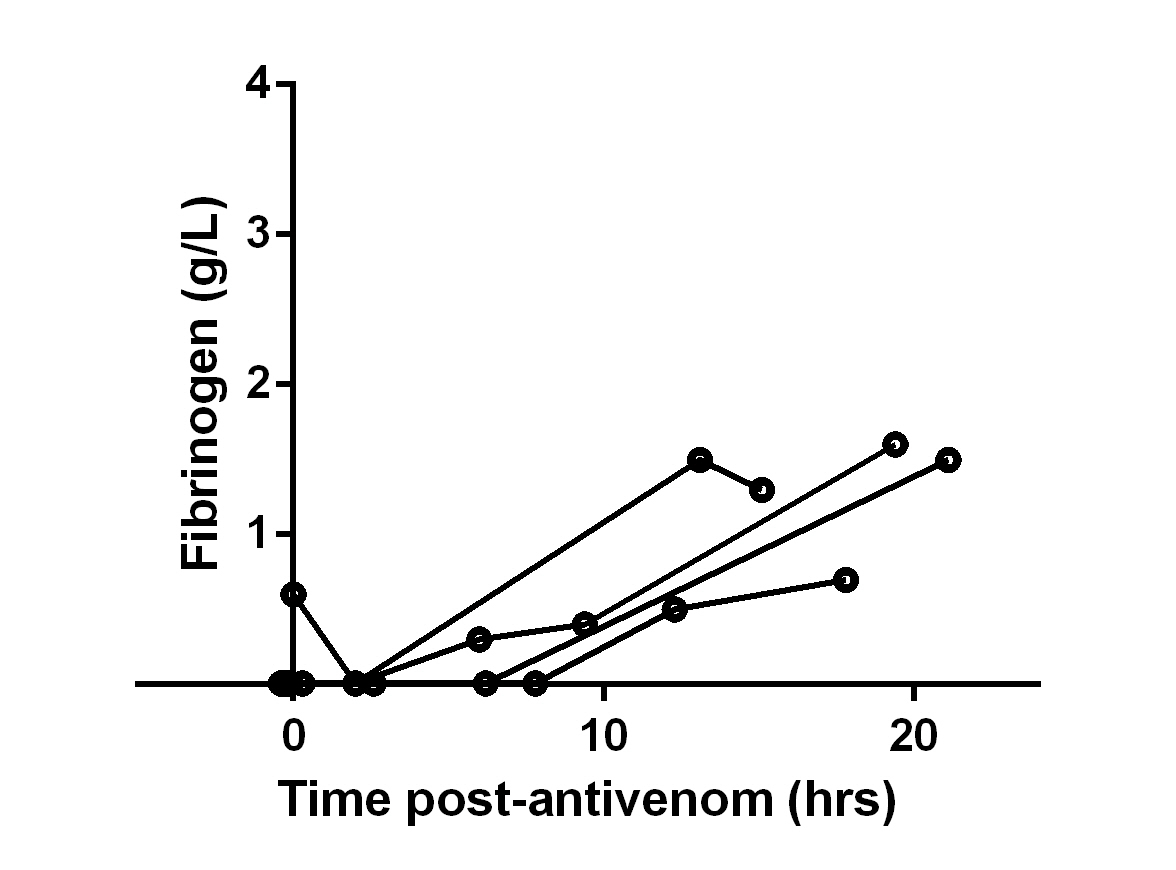

Supplement: S1 Figure — Fibrinogen levels (g/L) versus time in the two patients in the recurrence group and two patients in the non-recurrence group who had additional antivenom and were in the recovery phase of the coagulopathy. (JPG) [file pntd.0003304.s001.jpg]
